# Process evaluation of the Bristol girls dance project

**DOI:** 10.1186/s12889-016-3010-4

**Published:** 2016-04-21

**Authors:** S. J. Sebire, M. J. Edwards, J. M. Kesten, T. May, K. J. Banfield, E. L. Bird, K. Tomkinson, P. Blair, J. E. Powell, R. Jago

**Affiliations:** Centre for Exercise, Nutrition & Health Sciences, School for Policy Studies, University of Bristol, 8 Priory Road, Bristol, BS8 1TZ UK; Bristol Randomised Trials Collaboration, School of Social & Community Medicine, University of Bristol, Bristol, BS8 2PS UK; Health and Social Sciences, University of the West of England, Bristol, UK

**Keywords:** Physical activity intervention, Dance, Secondary school, Process evaluation, Adolescent, Girls

## Abstract

**Background:**

The Bristol Girls Dance Project was a cluster randomised controlled trial that aimed to increase objectively measured moderate-to-vigorous physical activity (MVPA) levels of Year 7 (age 11–12) girls through a dance-based after-school intervention. The intervention was delivered in nine schools and consisted of up to forty after-school dance sessions. This paper reports on the main findings from the detailed process evaluation that was conducted.

**Methods:**

Quantitative and qualitative data were collected from intervention schools. Dose and fidelity were reported by dance instructors at every session. Intervention dose was defined as attending two thirds of sessions and was measured by attendance registers. Fidelity to the intervention manual was reported by dance instructors. On four randomly-selected occasions, participants reported their perceived level of exertion and enjoyment. Reasons for non-attendance were self-reported at the end of the intervention. Semi-structured interviews were conducted with all dance instructors who delivered the intervention (*n =* 10) and school contacts (*n =* 9) in intervention schools. A focus group was conducted with girls who participated in each intervention school (*n =* 9).

**Results:**

The study did not affect girls’ MVPA. An average of 31.7 girls participated in each school, with 9.1 per school receiving the intervention dose. Mean attendance and instructors’ fidelity to the intervention manual decreased over time. The decline in attendance was largely attributed to extraneous factors common to after-school activities. Qualitative data suggest that the training and intervention manual were helpful to most instructors. Participant ratings of session enjoyment were high but perceived exertion was low, however, girls found parts of the intervention challenging.

**Conclusions:**

The intervention was enjoyed by participants. Attendance at the intervention sessions was low but typical of after-school activities. Participants reported that the intervention brought about numerous health and social benefits and improved their dance-based knowledge and skills. The intervention could be improved by reducing the number of girls allowed to participate in each school and providing longer and more in-depth training to those delivering the intervention.

**Trial registration:**

ISRCTN52882523 Registered 25th April 2013.

## Background

Physical activity (PA) during childhood is beneficial for physical and mental health [1–3]. A high proportion of young people [4] do not achieve the UK government’s recommendation of at least 60 min of moderate-to-vigorous intensity PA (MVPA) per day [5]. The transition between late childhood and early adolescence is a critical period of change during which PA declines [6, 7] for girls in particular [6], thus more research focussed on maintaining and increasing girls’ PA during this transition is needed.

Whilst schools can be an important setting in which to promote youth PA [8], promoting PA during the school day presents several difficulties such as limited curriculum time and competition for school facilities [9–11]. As such, non-curriculum after-school interventions offer an alternative means of promoting PA in schools [10, 11]. To date there have been limited rigorous, controlled trials of  after-school PA interventions [10].

Dance can be a high intensity activity that contributes towards meeting PA recommendations [12, 13]. It is a popular form of PA among adolescent girls in the UK [14] and is an enjoyable activity that provides an opportunity to socialise and learn new skills while being active [15]. Dance appeals to girls across socioeconomic status and is particularly successful in engaging those from deprived areas whom would normally drop out of PA during secondary education (11–16 years) [16]. Thus, delivering dance sessions during the after-school period could potentially help to increase adolescent girls’ PA.

We recently reported on the effectiveness of the Bristol Girls Dance Project (BGDP), known locally as Active7, a cluster randomised controlled trial [17]. The study aimed to determine the effectiveness of an after-school dance intervention on objectively-assessed (accelerometer) mean weekday minutes of MVPA among 11–12 year old girls. There was insufficient statistical evidence to suggest that the intervention was effective in increasing girls MVPA.

Alongside the trial we conducted a rigorous process evaluation to examine the processes underpinning the intervention which may help to explain its effects [18]. Process evaluations assess the implementation (i.e., intervention fidelity and dose), the process through which any change in outcomes may arise, and the context in which an intervention is delivered (which may influence the implementation and impact) [19]. A detailed process evaluation of the underpinning mechanisms can offer insight as to why an intervention was (in) effective [20]. Consistent with recent MRC guidelines [19], in this paper we report elements of the process evaluation related to intervention dose, attendance, session fidelity, session enjoyment and exertion. The influence of the school context (e.g., facilities, ethos, personnel, etc.) on intervention delivery has been considered in a separate paper [21]. In addition to this, a separate theory-based process evaluation paper has been published elsewhere exploring theoretical fidelity to self-determination theory (SDT) that underpinned the intervention [22] links to which will be posted on the project website (www.active-7.org).

## Methods

### Intervention design

The trial protocol has been published [23]. Briefly, BGDP was a two-armed, cluster randomised controlled trial in which 18 schools were randomised to either a control (*n =* 9) or intervention (*n =* 9) arm. All Year 7 girls (11–12 years) in recruited schools were offered a ‘taster’ dance session to experience the intervention. Up to 33 girls per school were recruited to the study. In total 571 girls participated (284 intervention and 287 control). Intervention schools received up to 40 dance sessions that included a range of dance styles, consisting of two 75 min after-school sessions per week between January and July 2014. The sessions were led by self-employed female dance instructors recruited to the study. Instructors attended a one day training session before the intervention, and a half day “booster session” mid-way through the intervention period led by an experienced dance instructor. At both training sessions instructors were trained (by SJS) in how to use the intervention manual and how to adopt a need-supportive teaching style in line with SDT [24, 25]. The training covered numerous issues related to expectations of the instructors, communication styles, practical activities to develop and use need-supportive teaching styles, and using effective behaviour management techniques. As the instructors were already practicing dancers, the content did not focus on any dance-specific skills but instructors had time to work together and share ideas and rehearse different choreography.

All instructors were given a *‘Guide for dance instructors’* to facilitate delivery of the intervention, which included plans for 40 sessions. The manual was developed by an expert dance teacher/teacher trainer and trialled in a pilot study [26]. The post-pilot study qualitative work led to improvements being made to the manual. The 40 session plans provided general guidance on structure, progression, content, and suggestions on how to facilitate a suitable motivational climate. The session plans became less detailed over the 40 sessions as the instructors were provided more freedom to base sessions on girls’ preferences and/or to work towards a developing a performance. Session plans included details on session aims, warm-ups, group activities, choreographed activities and cool-downs. Teaching points and strategies were also outlined. Instructors were able to use a variety of dance styles including but not limited to: modern, lindy hop, hip-hop, Charleston, street and musical theatre. They were encouraged to use a range of styles and involve the girls in choosing the style they would use. Dance instructors were able to use their professional experience to focus on the style they felt most comfortable teaching, but all instructors were competent teaching different styles and covered these throughout the project. The focus on a particular dance style lasted for approximately 10 sessions, but if a particular activity was not liked by the girls after several sessions, the focus would move to a new style.

### Data collection

#### Quantitative component

The process evaluation data relates to the intervention schools only. Participants were classified as receiving the intervention ‘dose’ if they attended at least two thirds of all sessions provided in their school. Dose was measured using attendance registers completed by dance instructors. At the end of the intervention, participants reported how true 13 reasons for non-attendance (e.g., “*I prefer to spend time with my friends*”) were for them on a 5-point scale (0 = *Not true for me* to 4 = *Very true for me*). An open ended question was included for girls to list other reasons for not attending. The 13 questions were based on a questionnaire that we have previously developed and used to assess attendance in extra-curricular programmes [27]. These data were obtained from 280 (99.6 %) girls in the intervention group, 84 girls gave ‘other’ reasons for not attending. Dance instructors self-reported fidelity to the intervention manual (‘*fully*’, ‘*partially*’ or ‘*not at all’*) for each session. To understand the receipt and impact of the intervention, participants in each school reported their perceived level of exertion [28] using a 10-point scale (0 = ‘*not at all tired’* to 10 = ‘*very very tired*’), and their enjoyment [29] using a 5-point scale (1 = ‘*not at all’* to 5 = ‘*a lot*’). These data were collected at the end of four randomly-selected sessions across the 40 sessions (i.e. one randomly-selected session between sessions 5–12, 13–20, 21–29 and 30–36).

Four intervention sessions per dance instructor were observed by study staff. The sessions were also audio recorded, with the recordings and observations being used to assess the need-supportive teaching strategies of dance instructors. The methods and findings are reported in a separate theory-based process evaluation paper [22].

#### Qualitative component

Semi-structured interviews (mean duration *=* 67.2 min, range = 41.4 to 91.4 min) were conducted with ten dance instructors who delivered the sessions in the intervention schools. Two instructors (one substitute instructor, and one instructor who delivered sessions in schools 21 and 51) each delivered half of the intervention sessions in one school (school 23). The interviews explored experiences of the intervention training, intervention fidelity, successes and challenges.

Semi-structured interviews (mean duration *=* 29.4 min, range = 22.1 to 38.4 min) were conducted with nine school personnel who were the main contact between the research team and the school (eight female, one male). School contacts discussed the logistics of the project including recruitment, intervention delivery, data collection, and areas for improvement. They also discussed factors that would affect disseminating the intervention on a larger scale.

A focus group was conducted with girls that received the intervention in each intervention school. Ten girls (including two reserves) per school were purposively selected to allow us to explore the views of girls from different tertiles of attendance (top tertile mean (SD) attendance = 27.8, 4.1; middle tertile = 17.1, 5.0; bottom tertile = 6.5, 1.7). To ensure that girls were able to share experiences of the intervention, girls who attended ≤3 sessions were excluded. 59 girls participated in the focus groups (*n =* 25, 16 & 18 high, moderate and low attenders respectively). Focus groups comprised girls from the different attendance tertiles, the size ranged from 3–8 participants and the mean duration was 42.4 min (range = 30.4-50.2 min). Focus groups explored factors that influenced participation, views on session content and delivery, views of the dance instructor and issues attached to wider roll-out of the BGDP.

All qualitative data were recorded using an encrypted digital recorder (Olympus DS-3500) and audio recordings were transcribed verbatim. Transcripts were anonymised and compared to the audio recordings to ensure accuracy. Written informed consent was obtained from all school contacts and dance instructors, with written parental consent obtained for children. The study was approved by the School for Policy Studies ethics committee at the University of Bristol (ref: Bristol Girls Dance Project). 

### Analysis

#### Quantitative data

Frequencies, percentages, means and standard deviations were calculated to describe recruitment, attendance, fidelity to the manual, reasons for non-attendance, exertion and enjoyment data.

#### Qualitative data

The Framework Method, a form of thematic analysis defined by the systematic production of a matrix that reduces data into a series of codes, was used to analyse the qualitative data [30]. Analysis was conducted by JMK, MJE, SJS, and TM. Following familiarisation with the transcripts through repeated reading, initial codes were created to summarise and interpret data. Inductively, the codes captured topics that emerged from the interviews. Deductively, the analysis probed data to understand whether the intervention was delivered in line with SDT [31]. A pre-defined ‘school context’ code was included to explore differences between schools (both SDT and school context are explored in separate papers). Initial codes were produced independently by team members who each coded three different transcripts (one dance instructor, school contact and focus group respectively). Codes were discussed in weekly meetings, iteratively refined and combined to produce three coding frameworks. The frameworks were applied to the remaining transcripts by JMK, MJE, and TM. Refinements were discussed at meetings and frameworks were amended as new information arose.

Coded data were inserted into a framework matrix in Nvivo (Version 10, QSR International Pty Ltd) to organise the data and help select illustrative quotes. To facilitate interpretation, a convergence coding matrix [32] was used to compare codes across the three informant groups to assess: ‘*agreement*’ (i.e., codes from more than one group agree), ‘*partial agreement’* (i.e., agreement between some but not all groups), ‘*silence*’ (i.e., code is found in one group but not others), and ‘*dissonance*’ (i.e., disagreement between informant group). Agreement was identified between informant groups in 22 (29 %) themes, partial agreement in 26 (34 %) themes, silence in 39 (51 %) themes and dissonance in 6 (7 %) of themes. JMK, MJE and TM double-coded two transcripts each, discussed them and agreed upon any discrepancies in interpretation. To ensure trustworthiness, four criteria were applied: credibility; transferability; dependability and confirmability (Table 1) [33]. Findings are presented in a mixed-methods format in which the main qualitative themes, supported with illustrative quotes, are interpreted in light of the quantitative data. All qualitative data are attributed to participants using the anonymised identification codes used during the study.

**Table 1:** Description of how the qualitative component addressed features of trustworthiness criteria

Trustworthiness feature	Description
Credibility (internal validity)	Familiarity and rapport between the interviewer (JMK), dance instructors and participants was developed over four visits to each school. By observing dance sessions an understanding of the content and delivery was established. This insight informed the refinement of interview guides and may have encouraged honesty in the interviews. Researcher bias in the selection of participants was minimised by a random selection of focus group participants by attendance. Views from all intervention schools were gathered. During analysis, frequent study team de-briefings ensured different interpretations of data were considered.
Transferability (external validity) and dependability (reliability)	Findings should be understood within the study context. However, if similar findings are elicited in different schools or interventions, this could demonstrate a degree of transferability. By providing in-depth details of the methods we ensure that the study is repeatable.
Confirmability (Objectivity)	Researchers (JMK, SJS, TM, MJE) worked to ensure that the findings reflected the experiences of participants. SJS and RJ developed the project and SJS uses SDT in his research. JMK attended four dance sessions within each school and became familiar with each school setting. Therefore this may have influenced her interpretation of qualitative information. TM did not perform any school visits and does not have a background in SDT. Therefore he was able to assume a role of checking that interpretations reflected the data.

## Results

Quantitative and qualitative results are presented alongside one another in two sections: 1) implementation and 2) receipt of intervention. The sub-sections contained within the two sections are detailed in Table 2.

**Table 2:** Categories of implementation and receipt of intervention in the Active 7 process evaluation

Implementation	Receipt of intervention
Intervention dose and attendance	Enjoyment
Understanding high attendance	Exertion
Reaching those who needed the intervention most	Perceived health, well-being, and psychological benefits
Impact of attendance on intervention delivery	Intentions to continue dancing
Dance instructor training	
Fidelity to the intervention manual	

### 1. Implementation

This section reports results related to intervention dance instructor training, dose, and the degree to which the session plan manual was adhered to.

### Dance instructor training

The majority of dance instructors thought that the training, along with their existing knowledge/expertise, adequately prepared them to deliver the intervention.

I think you kind of covered it from every angle (Dance instructor 32).

Bringing the group of instructors together led to an unanticipated but welcome creation of a peer-support network.

Although I knew some of [the other BGDP instructors] I didn't know some of them that well. So kind of learning more about them, and what they do, and what styles they’re interested in. And also, just kind of on a personal level, building that network as a freelancer, it can be quite isolating so that was quite nice to have that opportunity (Dance instructor 32).

Similarly, the mid-intervention booster training was viewed as an opportunity to reflect on the dance sessions delivered and an opportunity for peer sharing and learning.

It was quite reassuring. Even though it’s not nice to know that everyone else is having similar difficulties, it’s quite reassuring to think “actually, no, this is normal and people are having similar things or if not worse” (Dance instructor 32).

However, some practical elements of the induction training were considered inappropriate given the instructors’ experience. Also, some found the length of the ‘booster’ session to be too short.

In terms of the practical element, to be honest it’s, you know, the games and things are things I've been doing for the last 15 years (Dance instructor 42).

More time would have been useful. It felt quite rushed (Dance instructor 61).

### Intervention dose

All 40 dance sessions were delivered in four schools and between 37 and 39 sessions were delivered in the other schools. On average, 31.7 (range = 26–33) girls participated in the study in each school and 9.1 participants per school (range = 1−20) attended two thirds of all possible dance sessions.

**Fig. 1:**
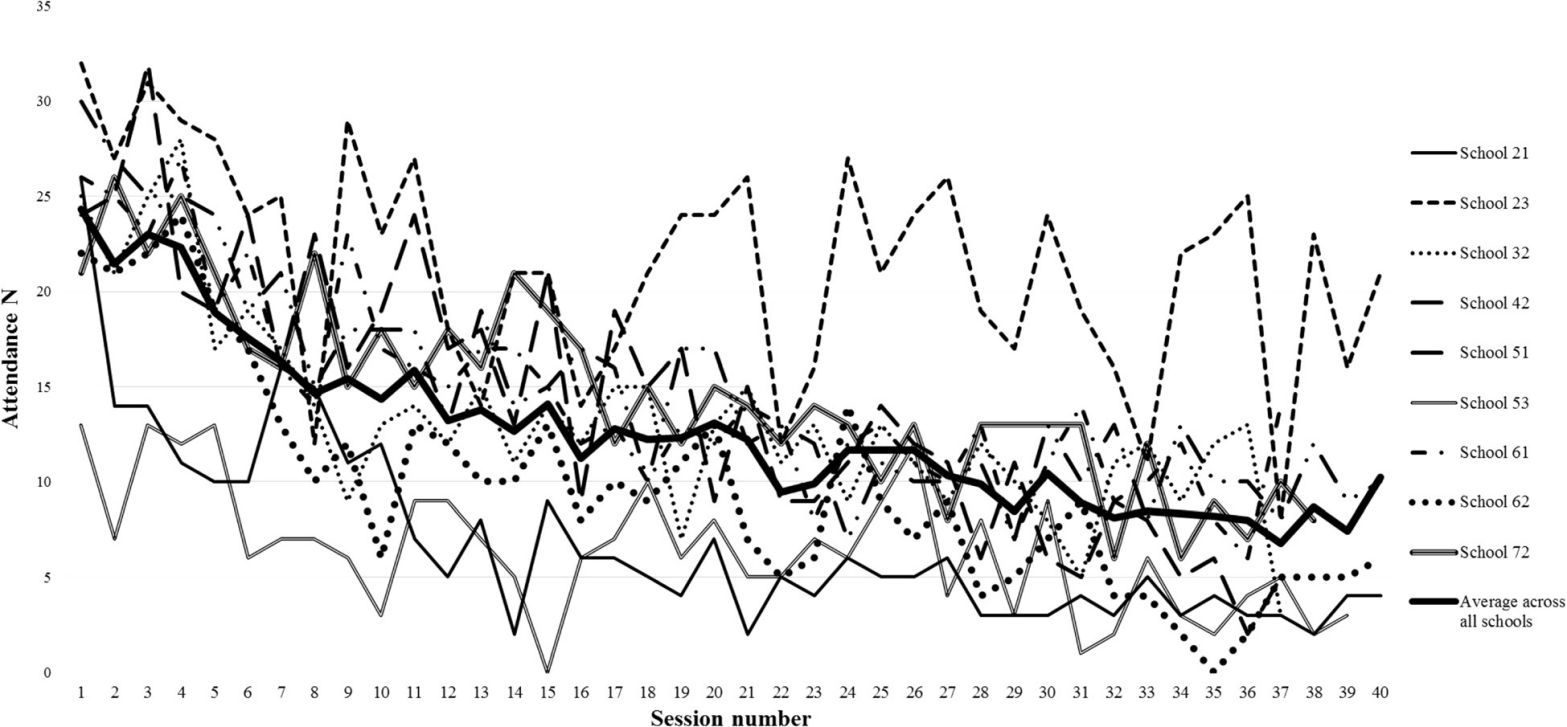
Attendance per dance session across all intervention schools

Figure 1 displays attendance by school over the course of the intervention. Mean attendance was 12.8 (SD = 7.0) girls per session (max = 32). Mean attendance at the first session was 24.3 (SD = 5.5) and steadily decreased to 10.3 (SD = 7.6) by the final session. School 23 had the highest and school 53 had the lowest average attendance. There was considerable variation in attendance between sessions in all schools and several sessions had zero attendance. One reason for this occurring was due to the school contact not informing the dance instructor that an alternative school-event was taking place (i.e., camp or sports day). 25 girls did not attended any sessions. 17 girls withdrew during the intervention (after attending only one session), whilst five girls withdrew from the study after attending some sessions (but did not provide data at any time points).

Whilst attendance was relatively low, some school contacts viewed the attrition rate as similar to other after-school clubs.

Everyone always starts like really enthusiastic… they’re very much like, “Oh, I’ll sign up for that” and then “I’ll just drop out half way through” (School contact 32).

That [decrease in attendance] was not a ‘dance thing’ or an ‘Active7’ thing, that’s just ‘a thing’ (School contact 62).

However, two school contacts suggested that the decline in attendance was notably high.

The attendance was horrendous. Really quite bad (School contact 42).

**Fig. 2:**
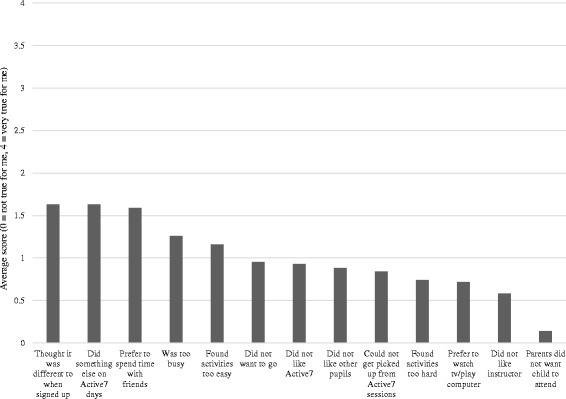
Self-reported reasons for not attending Active7 sessions

Girls self-reported the reasons why they did not attend some sessions (Fig. 2). While endorsement of all reasons was relatively low, the most common reasons were that participants had a different activity on the days Active7 ran, that sessions were not what they expected when they enrolled, and that they preferred spending time with other friends outside of the Active 7 dance class. For open responses, the most commonly cited reasons were ‘injury/illness/tired’ (*n =* 21), ‘issues with the dance project’ (*n =* 17), and ‘other sports clubs’ (*n =* 12).

### Understanding high attendance

In the school with the highest attendance (school 23), the dance instructor and school contact described the school catchment area as influencing attendance, attitudes to the project, and participant behaviour.

I think it’s just because the school's in a good area that the students are more … well-behaved, got better attendance (*Dance instructor 23).*

The type of students we’ve got in this school… they don’t want to let people down so I think they’ve got that in the back of their minds. They are aware that it’s a good opportunity for them, and they’ve got parental support so I think that’s a major impact (School contact 23).

The novelty of BGDP was also thought to partially explain the high attendance in school 23.

We haven’t really had something like this, like Active 7. That’s why loads of people started attending (Focus group 23).

One of the two dance instructors in this school thought the participants particularly valued their place in the project:

I felt like they wanted to stay in the project but they also understood that this was exclusive to them […] so I think they really valued their place in the class (Dance instructor 23).

### Reaching those who needed the intervention most

The intervention was seen to reach some girls who were perceived as in need of opportunities like BGDP due to low activity levels, limited dance experience, or financial barriers to participation.

It’s the quiet ones who are not making the school teams and so on, that’s benefitted them probably more than the really sporty ones (School contact 23).

My mum was just glad that something was actually free for once (Focus group 62).

The ones that were doing lots of things and that were naturally more talented didn't turn up which was interesting, but that means that things for people who are from broken families, who… have just transferred from another country… they perhaps are a bit oddballs and they come together in those situations and they feel at home which is nice (Dance instructor 53).

In contrast, for the school with the highest attendance, the dance instructor described the majority of girls as already attending several after-school activities.

A lot of the girls who I’m teaching are very sporty, go to dancing already, they’re not really the sort of key people that you’re looking for the project (Dance instructor 23).

For some girls, taking part in the BGDP replaced another form of PA.

I’d have been part of the [school] basketball team. That’s what I was doing before Active7. And now that it’s finished I’m going to join that again (Focus group 72).

### Impact of attendance on intervention delivery

Dance instructors found low attendance to be frustrating and some reflected personally on the decline in numbers.

I was quite angry, especially when I'd be sitting in the entrance and they’d just walk past me and not acknowledge me or say anything, it was really difficult to go in and … and be like ’hey, fun, ha-ha-ha!’ (Dance instructor 53).

Varying attendance resulted in the need to repeat the content of previous sessions to allow absent girls to keep up with the progressive building of dance pieces.

We were never able to complete anything […] I always had to produce something different every session because even when I had a couple of girls who were there all the time and every week, I could probably get them to teach it in a session afterwards, but after that they’d get bored of re-teaching it when there would be another new person at the next session (Dance instructor 53).

However, as attendance declined, the smaller groups of ‘committed’ participants were preferred by those attending and the instructor. This facilitated teaching and the formation of closer instructor-participant connections.

Quite a lot of people left, but actually in the last term when it was just the 15, 16, they were all incredibly committed […] and their energy in class was great so it was actually a lot better (Dance instructor 32).

Now there’s not that many people [in the sessions] it’s so much more relaxed and like everyone can just be themselves (Focus group 61).

### Fidelity to the intervention manual

**Fig. 3:**
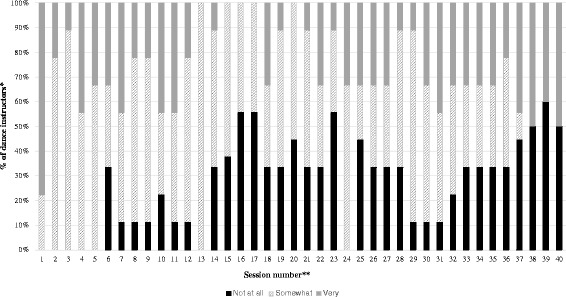
Fidelity to the intervention manual over the course of the intervention

Figure 3 shows instructors’ ratings of fidelity to the session plan manual. Overall 26.7 % of sessions delivered were reported as being ‘very much’ like the manual, 47.1 % were ‘somewhat’, and 25.9 % were rated ‘not at all’. It appears that instructors adhered to the manual most within the first five sessions and deviated from the manual more from session six onwards. All but two instructors (who rated 50 % & 76.9 % of sessions as ‘not at all’ like the manual), delivered the majority of their sessions ‘somewhat’ or ‘very much’ like that outlined in manual.

Manual adherence was discussed in the interviews. Generally the manual was regarded as a detailed, interesting and useful resource which encouraged participants to reflect on their progress.

I kept asking this to the girls-because it says in the manual a lot and I think it's nice-“oh, can you do that stretch a bit longer, have you noticed?” or “can you do that?” (Dance instructor 23).

However, the majority of instructors felt that given their level of training and experience the amount of detail was unnecessary.

When you've been teaching for 6, 7, 8 years… you’ve got that experience of working with groups beforehand and you know what works and you know what doesn’t work […] [the manual] could have maybe have been more… simplified and maybe, more suggestive (*Dance instructor 23).*

Furthermore, some content in the initial session-plans contradicted how the instructors would normally lead sessions which may partially explain the initial adherence to- followed by greater departure from the manual.

Where it went wrong for me was [when] trying to stick to the manual I maybe did things that near the beginning that I wouldn’t have done and that maybe set things up slightly against me in terms of managing behaviour (*Dance instructor 42).*

The dance instructors described using and adapting the session plans in various ways. Using the manual as a ‘guide’ and allowing participant input was cited by several instructors.

I used like what we were going to do etc. from [the manual] and then after that it was kind of … the children were more comfortable with me, I knew their technique strengths and it was kind of what I wanted to work on (Dance instructor 21&51).

In line with the finding that varied attendance disrupted session delivery, attendance and facility changes also disrupted adherence to the manual.

I kind of stopped reading [the manual] after a while because every session I had different kids, every session was in a different space or I couldn't get in a space. There was no way I could follow it (Dance instructor 53).

### 2. Receipt of the intervention

This section considers levels of enjoyment and exertion of participants and the qualitative perceptions of the impact on health, well-being and intentions to continue dancing.

### Enjoyment

**Fig. 4:**
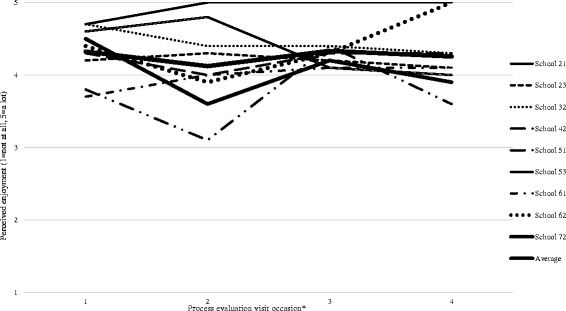
Mean perceived enjoyment per school during the intervention

Enjoyment of the dance sessions was high in the majority of schools throughout the intervention (mean *=* 4.3, SD = 0.3; range = 1 to 5) (Fig. 4). The qualitative findings support the quantitative data; group work, choreographing dance material and dancing to popular music were highlighted as particularly enjoyable.

It’s like another fun activity you can do with your friends (Focus group 42).

Different music every lesson, like recent music and stuff. So that made it like more fun because we like knew the songs and stuff (Focus group 23).

The dance instructors also felt that participants enjoyed creating new dance material.

We did a lot of choreography, because that’s what they really loved (Dance instructor 61).

Participants did not enjoy some dance styles, repetition of routines, and catching up to learn sequences from sessions they missed. The latter could be an explanation for decreasing attendance, as missed sessions may have led to a reluctance to attend future sessions when content has been missed.

I don’t know whether it’s the confidence thing or a lazy thing but they don” t […] want to try and catch up on what they” ve missed (Dance instructor 42).

I found [a particular style] quite boring. I enjoyed all the other ones… (Focus group 61).

### Exertion

**Fig. 5:**
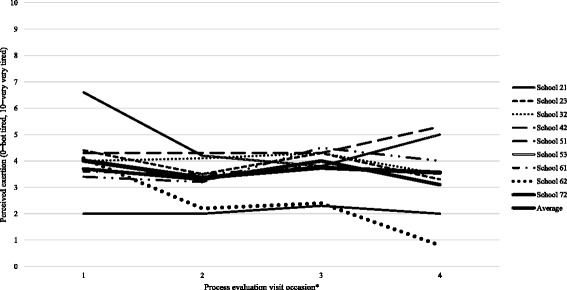
Mean perceived exertion levels per school on four occasions during the intervention

As shown in Fig. 5, ratings of perceived exertion were low (mean *=* 3.7, SD = 0.9) throughout the intervention with some variation within and between schools. However, the quantitative data did not align with the qualitative perceptions of pupil exertion reported by dance instructors and pupils which often referred to sessions as physically tiring.

Some were tiring and some were like kind of easy but like after any of them I kind of felt good about myself (Focus group 53).

I liked it a lot but I just got really tired, like physically (Focus group 32).

Three dance instructors’ views supported this perspective.

Quite a lot of them struggled with [some sessions], and I think that that’s mainly to do with fitness levels because they struggled with the pace of it rather than the actual movement (Dance instructor 61).

### Health, well-being, and psychological benefits

Participants in six schools reported various health benefits associated with participating in the study, including greater energy, fitness, flexibility and weight loss.

I couldn’t do press ups. Now I can […] I didn’t know how to and I sort of couldn’t. Now I can do them (*Focus group 53).*

Generally, girls believed that their confidence within dance and in non-dance settings increased, which was also observed by the instructors.

The fact that towards the end they wanted to do a different style each session, and they wanted to create their own bit each session, has got to be a good indicator on something like that […] It’s got to be a confidence thing (Dance instructor 62).

It like got me a bit more confident around my friends because usually I wouldn’t really do like dancing (Focus group 42).

### Intentions to continue dancing

One school contact suggested that the intervention increased the likelihood that girls would continue dancing within the curriculum. Six dance instructors communicated the girls’ interest in continuing with BGDP into Year 8.

Half of them are taking dance next year and I don’t think, you know, some of them wouldn’t have said that was even an option at the start of the year that they were taking, so it has had an impact on those girls that have stayed (School contact 61).

[Participants] were already asking ’okay, so are you coming back next year? It could be Active8–Activate! (Dance instructor 21).

## Discussion

This paper presents the findings of the BGDP process evaluation. By exploring the effects of dance on physical activity levels, the BGDP built on previous dance-based interventions among adolescent girls which have shown positive effects on psychosocial outcomes such as self-esteem [34] and physical self-perceptions [35]. The detailed process evaluation of the BGDP provides a new view on the workings of such interventions which can be used to understand why it did not increase girls’ physical activity as hypothesised and inform future intervention design. Average attendance at the BGDP sessions declined between the first and final session, with mean attendance falling from 24.3 (77.11 %) initially to 10.3 (26.06 %) in the final session. The BGDP feasibility trial (nine weeks in duration) reported a decrease in attendance, although the decline was less steep (from approximately 90 % initially to 60 % by the final session) [26]. The qualitative findings suggested that the decline in attendance was typical of after-school interventions but higher than dance instructors’ regular (often fee-paying) sessions. It is possible that girls who enrol in fee-paying dance sessions feel more competent in dance upon enrolling and have a greater sense of intention or perceived obligation to attend than girls in a less formal extra-curricular environment who may sign up to try a new activity in a free and safe environment. While the latter is highly desirable, more work is needed to understand how to retain those girls in the programme. It is important to note that only one school achieved maximum attendance at the first session. A decline in attendance can therefore only be partially explained by the experience of the intervention. As such, efforts are required to understand how to encourage those who sign up to after-school activities to attend initially. Participant drop-out and variability in attendance has been recorded in other PA interventions involving young people [36–38]. A number of previous PA intervention studies have reported declining and/or fluctuating attendance, alongside high enjoyment ratings [27, 37, 39]. For example, attendance in the ACT trial ranged from 40-51 % [40]. It has been suggested that parental support and transportation is pivotal to maintaining high attendance [41] and contacting parents of children who had poor attendance has previously resulted in small improvements in attendance [40].

The decline in attendance was not perceived as uniformly negative as all respondent groups suggested that both the quality of sessions and group cohesion increased as attendance declined. Girls who continued attending believed that their experience improved within the smaller group. Smaller intervention group sizes may be favourable as they create an optimal learning climate in which participants can have fun and enjoy themselves [42, 43]. Conversely larger groups have been found to adversely affect group dynamics and lead to poor behaviour [36, 38]. While future interventions could consider reducing the initial cohort size to create a committed smaller group, it may be that within school settings, larger initial groups are needed to allow for smaller groups to arise from natural attrition. Additionally, smaller, more exclusive groups, may not be appropriate in a school setting aimed at providing opportunities for all children.

Enjoyment of the intervention sessions was high. However, enjoyment was only rated by girls who attended the dance session on the day enjoyment was measured. While this accurately reflects the high enjoyment of the girls who were retained in the intervention, it does not reflect the views of those who dropped out (potentially because they did not enjoy the sessions) and thus may have inflated perceptions of enjoyment. However, the reasons girls gave for not attending did not align with factors seemingly associated with enjoyment, but reflected competing commitments, social preferences and the sessions not matching their expectations. Similarly, competition with alternative commitments and responsibilities was the most prominent reason for non-attendance in previous child-focussed PA interventions [27, 38].

Girls’ perceived levels of exertion during the dance sessions were low. This echoes the findings of the BGDP pilot study, in which exertion was 3.5 out of 10 [26]. Jago et al. [44] reported mean exertion levels of 5.9 out of 10 [38] for a four week Pilates intervention for 11 year old girls. Similarly, a US study found that levels of child PA in dance session were low [45]. In this study, anecdotal experiences of researchers attending the dance sessions to collect data indicated, alongside the qualitative reports of girls and dance instructors, that girls were exerting themselves considerably. The inconsistency of these findings could be due to girls misunderstanding the scale or that the measure lacks validity in this population group and in an after-school PA setting. Whilst the individual session plans aimed to provide within-session MVPA, in future studies which have a similar intervention design, more emphasis could be placed on optimising the intensity of within-session activity [17]. This may require the selection of certain dance styles that have been found to foster higher levels of MVPA (e.g., jazz, tap) [46]. However, it is important to balance this focus on activity intensity with allowing participants to choose dance styles that they enjoy and that facilitate the progressive building of their competence.

Fidelity to the intervention session manual varied between instructors and over the course of the intervention and the levels of fidelity in this study appear to be slightly lower than that of others [36] [39]. The majority of instructors used the manual to guide the initial sessions but progressively deviated from the session plans because: (a) as they became more familiar with the pupils, they were able to tailor sessions to their preferences and skills which may not have matched the session plans and (b) the inconsistent pupil attendance prevented dance instructors from delivering the manual in the intended sequence.

The relevance of the dance instructor training and perceived use of the session plan manual appeared to be affected by dance instructor experience. All instructors found elements of the training and manual to be informative and key successes of the training included the formation of a peer-support network and the mid-intervention booster session. Sharing ideas and experiences related to programme delivery was valued by instructors and is a strategy that has been used by Hall et al. [47], where dance instructors reported wanting a longer booster session to optimise sharing of best-practice. For some, however, the training content was considered to be too basic. Providing training for a diverse group of intervention deliverers will inevitably lead to insufficient coverage for some, however it is vital that all who deliver interventions are provided with the same information and guidance in order to ensure consistency across intervention sites. Commensurate with our findings, Guagliano et al. [48] found that experienced sports coaches relied on their knowledge rather than intervention materials when delivering training sessions. While such experience could result in effective innovation within an intervention, fidelity could be at risk if experience-led changes to the content delivered or style of delivery are at odds with the planned intervention.

### Strengths and limitations

This paper provides an in-depth, mixed-methods process evaluation of the BGDP intervention assessed from the perspectives of multiple stakeholders (i.e., participants, implementers and facilitators). The qualitative data were analysed before the outcome data to avoid bias in interpretation [49]. A school contact in all intervention schools was interviewed, as were all dance instructors who delivered the intervention. Researcher bias in the selection of focus group participants was minimised by the random selection of participants from different attendance tertiles. An in-depth description of how the research addressed published trustworthiness criteria is presented in Table 1.

This study has several limitations. Although we interviewed girls, school contacts and dance instructors, it may have been useful to explore the perceptions of parents, particularly with regards to issues surrounding attendance. Furthermore, some process evaluation components are subject to social desirability bias in which responders may report what they think the researcher wants to hear. This may be true of the interviews, reports of adherence to the manual, and the measures of enjoyment and exertion.

## Conclusions

The data presented in this paper show that, although the BGDP did not increase girls’ PA, dance-based after-school interventions can have a positive qualitative impact on participants. Girls enjoyed the intervention and identified health and social benefits of taking part. Attendance was relatively low and declined over time, however absence was largely the result of competing activities (as opposed to a dislike of the intervention). The intervention could be improved by having smaller groups, with a greater emphasis on encouraging consistent attendance. This may improve participants' experiences, reduce the need for repetition, and facilitate faster skill progression. Collaborating with dance instructors who are at different stages of their career to refine the session plan manual may improve the appropriateness of the manual for instructors with a range of abilities and thus increase fidelity. Additionally, a longer ‘booster’ session for instructors, mid-way through the intervention, may provide greater opportunity to discuss problems and resolve ongoing concerns.

### Ethics and Consent to Participate statement

Written informed consent was obtained from all school contacts and dance instructors, with written parental consent obtained for children. The study was approved by the School for Policy Studies ethics committee at the University of Bristol (ref: Bristol Girls Dance Project). Written parent consent was obtained for all children who wished to participate in the study.

### Consent to publish statement

Not Applicable.

### Availability of data and materials

The dataset (s) supporting the conclusions of this article are not currently available.

## Abbreviations

BGDP: Bristol girls dance project
MRC: medical research council
MVPA: moderate-to-vigorous physical activity
PA: physical activity
SDT: self-determination theory
